# Post-stroke fatigue and its correlation with family functioning in patients who have experienced a first episode of stroke

**DOI:** 10.3389/fnagi.2024.1440163

**Published:** 2024-10-21

**Authors:** Ruhuang Zhu, Haiping Huang, Yueting Yu, Shaorui Bao, Na Lin, Meichun Shu

**Affiliations:** ^1^Department of Neurology Nursing Unit Ward 17, The First Affiliated Hospital of Wenzhou Medical University, Wenzhou, Zhejiang, China; ^2^Department of Neurology Nursing Unit Ward 361, The First Affiliated Hospital of Wenzhou Medical University, Wenzhou, Zhejiang, China; ^3^Department of Neurology Nursing Unit Ward 362, The First Affiliated Hospital of Wenzhou Medical University, Wenzhou, Zhejiang, China

**Keywords:** stroke, post-stroke fatigue, family functioning, clinical care, fatigue severity scale

## Abstract

**Objective:**

This study aimed to analyse the relevant factors that may affect post-stroke fatigue (PSF) in patients with stroke and further explore the correlation between family functioning and PSF.

**Methods:**

Patients who had experienced a first episode of stroke and were admitted to the Department of Neurology of the First Affiliated Hospital of Wenzhou Medical University were rigorously screened. The general data and family functioning of the patients on admission were collected, and their family adaptation, partnership, growth, affection and resolve scores and their PSF on the 5th day of admission were collected using the fatigue severity scale (FSS). Multiple linear regression analysis was then utilized to explore the factors affecting PSF in patients with stroke.

**Results:**

A total of 220 questionnaires were distributed, and 220 were returned, with 212 valid questionnaires and a valid return rate of 96.4%. These 212 patients had a family functioning score of 6.58 ± 2.00 and an FSS score of 36.62 ± 10.96. Spearman’s correlation analysis showed negative correlations between the FSS scores and the adaptation, partnership, growth, affection, resolve and family functioning scores (*r* = −0.380, −0.505, −0.470, −0.303, −0.281 and −0.712, respectively; *p* < 0.001). Furthermore, multiple linear regression analysis showed that family functioning (β′ = −0.516), marital status (β′ = −0.244), household income (β′ = −0.185), literacy (β′ = −0.181) and activities of daily living (β′ = −0.084) were influential factors for PSF in patients with stroke (*p* < 0.05).

**Conclusion:**

There is a significant negative correlation between family functioning and PSF, suggesting that better family functioning may help mitigate the severity of post-stroke fatigue. Healthcare providers should identify interventions to help patients and families address fatigue, boost disease recovery, promote patients’ physical and mental health and improve their quality of life.

## 1 Introduction

Stroke is the second leading cause of death and is a major contributor to disability worldwide (Campbell and Khatri, 2020). Epidemiology studies have revealed a substantial increase in annual stroke incidence and deaths between 1990 and 2019 despite a significant decrease in age-standardized rates of stroke, particularly among those aged > 70 years (GBD 2019 Stroke Collaborators, 2021; Wu et al., 2019). Rehabilitation after stroke is a complex process that includes the recovery of physical function, improvement of cognitive function and psychosocial adaptation. However, post-stroke fatigue (PSF) has been shown in recent years to be a common and serious sequela affecting patients’ recovery and quality of life (Lanctôt et al., 2020; Vincent-Onabajo and Adamu, 2014). Fatigue, a subjective feeling, is often described as persistent physical and/or mental exhaustion that goes beyond the usual sense of tiredness. Post-stroke fatigue is characterized by persistent physical, cognitive and/or emotional fatigue, which severely affects the patient’s daily life and functioning. It is a symptom that frequently occurs in patients after the occurrence of stroke and causes them to have painful feelings, manifesting as both physical pain and emotional distress. The number of patients with stroke in China is increasing with the aging of the population, although, in recent years, the incidence of stroke in younger people has increased. This has led to the growing challenge of achieving an improved and faster recovery among patients after stroke. Family is of paramount importance for patients with stroke, as 80% of their care is provided by the family during the acute phase of their illness (World Health Organization, 2011). A family is viewed as a system with an efficacy that is reflected in the ability its members to interact, communicate emotionally with each other and respond together to stressful events (Juárez-Belaúnde et al., 2024). Family functioning refers to the interrelationships, communication and collaboration among family members and their ability to cope with stress and adversity. Family functioning may be subject to a variety of factors including, but not limited to, familial support, family dynamics and the mental health status of family members. Olson’s family systems theory (Juárez-Belaúnde et al., 2024) suggests that it is an essential function of the family to assist family members with chronic diseases to improve their health behaviors (Yahav, 2002; Franklin and Streeter, 1993). Favorable family functioning promotes the implementation of healthy behaviors in patients and enhances their ability to cope with the disease, which is of great significance for disease recovery (Krauth, 2017; Pesantes et al., 2018). These findings underscore the importance of family functioning in promoting effective coping mechanisms and enhancing recovery in patients with chronic diseases, including stroke (Dunbar et al., 2008; Trief et al., 2006). Although limited, there is emerging literature suggesting a potential correlation between family functioning and post-stroke fatigue (PSF). Several studies have demonstrated that family dynamics, such as communication, emotional support, and caregiving roles, play a significant role in the recovery process for stroke patients, which may indirectly influence PSF. For example, studies have shown that higher levels of family cohesion and support are associated with improved recovery outcomes and lower fatigue levels in stroke survivors (Dunbar et al., 2016; Indelicato et al., 2020). In the present study, we investigate the effect of family functioning on PSF in patients in the early stages of stroke by surveying the current status of PSF to provide theoretical support for clinical care.

## 2 Participants and methods

### 2.1 Participants

To ensure the study had sufficient power to detect the anticipated correlations between family functioning and PSF, the required sample size was calculated. Based on a hypothesized correlation coefficient (*r*) of 0.3, a power of 80% and a significance level (α) of 0.05, it was determined that 85 participants were necessary. Accounting for an anticipated dropout rate of 10%, this figure was adjusted to 95 participants to maintain statistical integrity throughout the study’s duration. The formula used to calculate the sample size for correlation studies, which factors in these parameters, was derived from Cohen’s statistical power analysis for the behavioral sciences (Chase and Chase, 1976).

Patients with a first episode of stroke admitted to the Department of Neurology of the First Affiliated Hospital of Wenzhou Medical University between October 2021 and July 2022 were rigorously screened using convenience sampling; this method was chosen because of time and resource constraints and in consideration of the fact that stroke is a specific disease. The inclusion criteria were as follows: patients whose clinical manifestations met the diagnostic criteria for stroke (Campbell et al., 2019) and whose stroke was confirmed through imaging (cranial computed tomography or cranial magnetic resonance imaging); those with a first episode of stroke and in a stable condition; those who were conscious and able to cooperate with the investigation; and those who gave informed consent and volunteered to participate in the study. The inclusion criteria were as follows: patients whose clinical manifestations met the diagnostic criteria for stroke (Campbell et al., 2019), confirmed through imaging (cranial computed tomography or cranial magnetic resonance imaging); those with a first episode of stroke and in stable condition; those who were conscious and able to cooperate with the investigation; and those who gave informed consent and volunteered to participate in the study. The exclusion criteria were as follows: patients with severe aphasia; those with severe cognitive impairment; those with malignant tumors; those with degenerative diseases of the central nervous system such as Parkinson’s disease; those with a previous history of mental illness; those with other recent major stressful life events such as divorce or the death of a loved one; and those with pre-stroke fatigue (PrSF). Patients with PrSF were excluded to isolate the stroke’s direct impact on fatigue levels. Pre-stroke fatigue was assessed using the fatigue severity scale (FSS), with a cut-off score set above the clinical threshold to identify significant fatigue. A total of 15 individuals meeting these criteria were excluded.

This study was conducted in accordance with the Declaration of Helsinki and approved by the Ethics Committee of the First Affiliated Hospital of Wenzhou Medical University (Approval number: 2022171). An informed consent form was signed by all participants in this study.

### 2.2 Methods

#### 2.2.1 Survey tools

##### 2.2.1.1 General patient data

All enrolled participants completed a general data questionnaire. The questionnaire was designed specifically to investigate the type of disease, gender, age, marital status, education level, household monthly income per capita (referred to as household income), medical payment methods, employment, living arrangement, activities of daily living (ADLs), number of comorbid chronic diseases, smoking history and alcohol history of the participants. The ADLs were measured using the modified Barthel index (MBI) (Wang et al., 2023), a tool most commonly used to assess the ability of patients with stroke to perform ADLs. The measurement consists of 10 items, namely feeding, bed-to-wheelchair transfer, personal hygiene, toileting, bathing, walking, walking up and down stairs, dressing and bowel control, with a total score of 100. The lower the MBI score is, the more severe the dysfunction and the greater the dependence. The degree of dependence was categorized into four grades, with ≤ 40 points categorized as severe dependence (all items required care from others – poor), 41–60 points categorized as moderate dependence (most items required care from others – fair) and 61–90 points and 90–100 points (Juárez-Belaúnde et al., 2024), categorized as mild or no dependence, respectively (few or no items required care from others – good). These categories were based on established scales, as reported (Duffy et al., 2013). Smoking was defined as an average of at least one cigarette per day for > 1 year; alcohol consumption was defined as an average of at least 50 ml of alcoholic beverages with a standard alcohol content (or other liquors of equivalent alcoholic content) per day for > 1 year.

##### 2.2.1.2 Family adaptation, partnership, growth, affection and resolve score

The family adaptation, partnership, growth, affection and resolve (APGAR) score, designed by Smilkstein et al. (1982) in 1978 and based on family functioning characteristics, was applied to assess patients’ satisfaction with family functioning. It was developed to measure the five dimensions of adaptation, partnership, growth, affection and resolve in five separate questions. Each question was assigned a score of 0–2, with a total score of 0–10 points. The degree of family dysfunction was based on the total score, with 7–10 points defined as good family function, 4–6 points as moderate family dysfunction and 0–3 points as severe family dysfunction, with a Cronbach’s α coefficient of 0.94 (Liu et al., 2015). The family APGAR score delivers an assessment of familial support and functioning from a medical perspective and is now widely used in patients with cerebrovascular disease (Mei et al., 2023).

##### 2.2.1.3 Post-stroke fatigue

Post-stroke fatigue was scored using the FSS (Lenaert et al., 2022), which is mainly designed to assess the severity of physical and mental fatigue felt by patients as a result of the disease. When assessing patients with stroke using the FSS, some scholars have diagnosed a score of ≥ 36 points as indicative of PSF (Wu and Wang, 2007). The FSS is comprised of a total of nine entries, each of which is scored on a 7-point Likert scale ranging from 1 to 7. A score of 1 indicates a ‘strongly disagree’ response to the corresponding entry, and a score of 7 represents a ‘strongly agree’ response to the corresponding entry. The final FSS score for the patient was obtained by summing the total score of the nine entries. The higher the score is, the more severe the patient’s fatigue (Zhang et al., 2021). The FSS score was highly reliable, with a Cronbach’s α coefficient of 0.90.

##### 2.2.1.4 Sleep disturbances

The Athens Insomnia Scale (AIS) was used to measure the sleep quality of patients during the study period (Soldatos et al., 2000). The AIS is an internationally recognized self-assessment scale for sleep quality, consisting of eight items. The scale is a 4-point Likert scale, scored 0, 1, 2 or 3, with 0 representing ‘not at all’ and 3 representing a ‘severe’ impact. The total score is 24 points. Scores ranging from 0 to 4 indicate no sleep disturbances, 4–6 suggest possible insomnia and scores above 6 represent insomnia. The higher the score is, the poorer the sleep quality and the more severe the sleep problems. The Cronbach’s alpha coefficient for this scale is 0.875.

##### 2.2.1.5 Anxiety and depression

The Hospital Anxiety and Depression Scale was developed to screen anxiety and depression in hospital patients (Cassiani-Miranda et al., 2022). It is composed of 14 items divided into two subscales: the depression subscale (7 items) and the anxiety subscale (7 items). Scores from 0 to 7 indicate no symptoms, 8 to 10 suggest possible symptoms and 11 to 21 indicate definite symptoms. The overall Cronbach’s alpha coefficient for the scale, as well as those of the anxiety and depression subscales, are 0.88 and 0.81, respectively.

#### 2.2.2 Data collection

Patients were screened at the time of admission and assessed for family functioning after enrolment. A fatigue severity survey was conducted on the 5th day of enrolment, and all patients gave informed consent to be surveyed face-to-face by the investigator. In addition, the case data of patients suffering a first episode of stroke were retrieved with the cooperation of medical workers in the department. During the survey, the content of the questionnaire was first interpreted in detail, and then the requirements for completing the questionnaire were explained to ensure that the respondents understood it completely and could complete the questionnaire independently. Patients with dyslexia or those who were unable to complete the questionnaire by themselves due to hemiplegia completed the questionnaire one by one according to their circumstances after the investigator had read the questionnaire to them to make sure that they understood it completely. After the interviews, the questionnaires were checked and returned immediately to the investigator.

#### 2.2.3 Quality control

The questionnaire survey was performed by two nurse practitioners and three charge nurses in the Department of Neurology who were uniformly trained and communicated using the same terminology. Before the survey, the investigator was required to explain the knowledge of stroke-related illnesses to facilitate favorable contact and communication with the patients. During the survey, the patients were selected in strict accordance with the inclusion and exclusion criteria. When communicating with the patients, unified guidance language was used to explain the items that patients did not understand objectively and accurately. Patients completed the questionnaires face-to-face with the investigator answering independently and without hints; the questionnaires were then checked and returned immediately. Questionnaires, if completed by the investigator instead of the patient, were verified with the patient after completion. At the entry stage, the questionnaires were rechecked and entered into the computer after ensuring that there were no errors, and qualifying questionnaires were numbered. A double-checking entry method was used to avoid human-generated data errors, and all data were entered into an electronic database and statistically analysed.

### 2.3 Statistical analysis

All valid raw data were numbered and managed using Excel software (Microsoft Corporation, Redmond, WA, USA). Count data, such as general clinical and sociological data and scores of each scale, were expressed as percentages and frequencies, and those conforming to normal distribution were expressed as mean ± standard deviation. For quantitative data with a non-normal distribution, the distribution was described using the median (first quartile, third quartile) (M[P25, P75]). The Kruskal–Wallis H test or Mann–Whitney U test was used to compare differences between groups. For multiple groups where the difference was statistically significant, the Mann–Whitney U test was used for pairwise comparisons between groups, and the Bonferroni method was used to correct the test results. Multiple linear regression analysis was utilized to explore the relationship between the variables of age, family income, marital status and literacy and PSF in patients who have experienced a first episode of stroke. Furthermore, Spearman’s correlation analysis was employed to analyse the potential correlation between family functioning and PSF in patients who have experienced a first episode of stroke. All data in this study were processed using the SPSS 25.0 data statistical software package (IBM Corporation, Armonk, NY, USA), with α = 0.05 as the test level.

## 3 Results

### 3.1 General data and disease-related characteristics of patients

**FIGURE 1:**
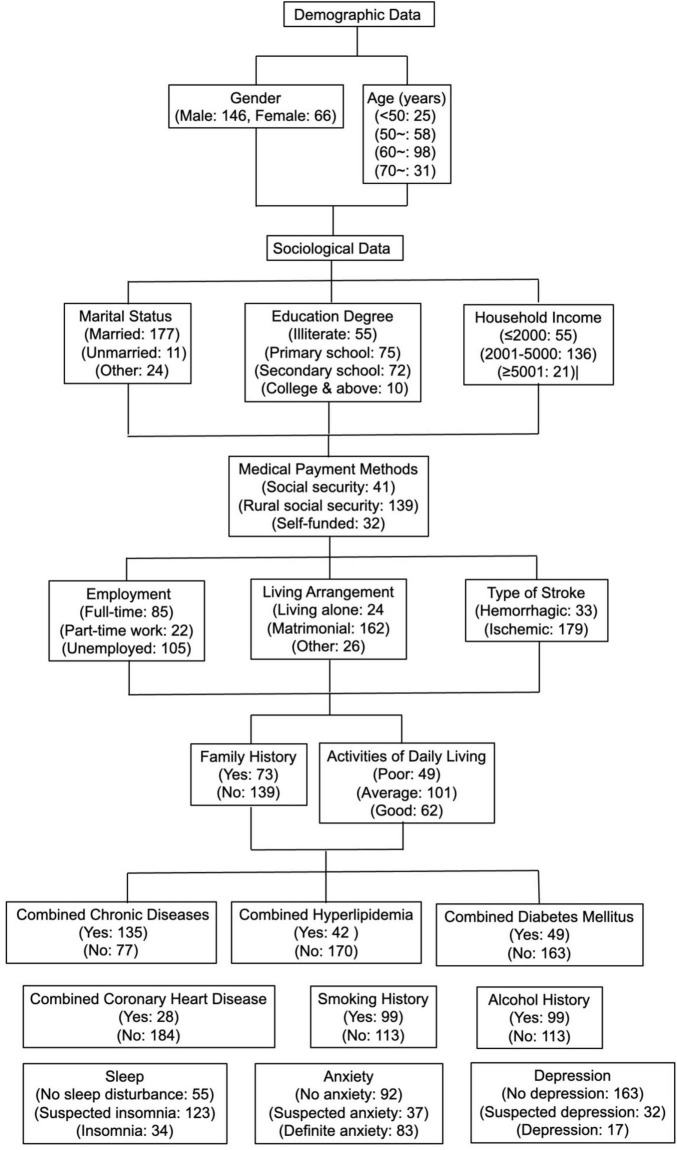
Flow chart of general demographic sociological and disease-related data of 212 first-episode stroke patients.

A total of 220 questionnaires were distributed, and 220 were returned, with a recovery rate of 100.0%. Eight invalid questionnaires were excluded, and 212 valid questionnaires were included, with a valid recovery rate of 96.4%. See Figure 1 and Table 1 for details.

**TABLE 1:** General demographic sociological and disease-related data of 212 first-episode stroke patients.

Item	Grouping	Number of cases (*n*)	Composition ratio (%)
Gender	Male	146	68.9
	Female	66	31.1
Age (years)	<50	25	11.8
	50∼	58	27.4
	60∼	98	46.2
	70∼	31	14.6
Marital status	Married	177	83.5
	Unmarried	11	5.2
	Other	24	11.3
Education degree	Illiterate	55	25.9
	Primary school	75	35.4
	Secondary school	72	34.0
	College and above	10	4.7
Household income (RMB)	≤2000	55	25.9
	2001–5000	136	64.2
	≥5001	21	9.9
Medical payment methods	Social security	41	19.3
	Rural social security	139	65.6
	Self-funded	32	15.1
Employment	Full-time	85	40.1
	Part-time work	22	10.4
	Unemployed	105	49.5
Living arrangement	Living alone	24	11.3
	Matrimonial cohabitation	162	76.4
	Other	26	12.3
Type of stroke	Hemorrhagic stroke	33	15.6
	Ischemic stroke	179	84.4
Family history	Yes	73	34.4
	No	139	65.6
Activities of daily living	Poor	49	23.1
	Average	101	47.6
	Good	62	29.3
Combined hypertension	Yes	135	63.7
	No	77	36.3
Combined hyperlipidemia	Yes	42	19.8
	No	170	80.2
Combined diabetes mellitus	Yes	49	23.1
	No	163	76.9
Combined coronary heart disease	Yes	28	13.2
	No	184	86.8
Smoking history	Yes	99	46.7
	No	113	53.3
Alcohol history	Yes	99	46.7
	No	113	53.3
Sleep	No sleep disturbance	55	26.0
	Suspected insomnia	123	58.0
	Insomnia	34	16.0
Anxiety	No anxiety	92	43.4
	Suspected anxiety	37	17.4
	Definite anxiety	83	39.2
Depression	No depression	163	76.9
	Suspected depression	32	15.1
	Definite depression	17	8.0

### 3.2 Status and comparison of post-stroke fatigue in patients with stroke

#### 3.2.1 Status of post-stroke fatigue in patients with stroke

The FSS scores of the 212 patients ranged from 14 to 60, with a mean of 36.62 ± 10.96. They were categorized into two groups: 102 (48.1%) patients had PSF (the ‘fatigue group’), and 110 (51.9%) patients had no fatigue (the “non-fatigue group”). In the fatigue group, there were 64 men and 38 women, with a mean age of 64.2 ± 8.3 years; in the non-fatigue group, there were 82 men and 28 women, with a mean age of 57.2 ± 8.9 years. No statistically significant association was found between the two groups in terms of gender (*χ^2^* = 3.437, *p* = 0.063); however, there was a statistically significant difference in terms of age comparison (*t* = 5.937, *p* < 0.001, mean difference = 7.007).

#### 3.2.2 Comparison of fatigue severity scale scores among patients with different clinical data

No statistically significant difference was found in the comparison of FSS scores for stroke type, family history, types of comorbid chronic diseases, smoking history and alcohol history (*p* > 0.05). However, there was a statistically significant difference in the comparison of FSS scores for gender, age, marital status, education level, household income, medical payment methods, employment, living arrangements, ADLs, sleep, anxiety and depression (*p* < 0.05, see Table 2).

**TABLE 2:** Comparison of FSS scores among those with different clinical data M(P_25_,P_75_).

Clinical data	Number of cases	FSS score M(P_25_, P_75_)	Z(H) value	*P*-value
Gender			2.747	0.006
Male	146	32 (26, 45)		
Female	66	42 (31, 46)		
Age (years)			28.173a	<0.001
<50	25	30 (27, 42.5)		
50–59	58	29.5 (25, 33)		
60–69	98	41.5 (27, 50)		
70–80	31	43 (35, 49)		
Marital status			63.073a	<0.001
Married	177	31 (26, 43)		
Unmarried	11	50 (48, 54)		
Other	24	52 (49, 54.7)		
Education degree			102.213a	<0.001
Illiterate	55	50 (46, 53)		
Primary school	75	38 (27, 44)		
Secondary school	72	27.5 (25, 32)		
College and above	10	25.5 (21.3, 30.3)		
Household income (RMB)			50.432a	<0.001
≤2000	55	46 (21, 51)		
2001–5000	136	31 (26.3, 43)		
≥5001	21	30 (22, 31)		
Medical payment methods			24.524a	<0.001
Social security	41	29 (25.5, 33.5)		
Rural social security	139	41 (29, 48)		
Self-funded	32	31 (24, 45)		
Employment			29.540a	<0.001
Full-time	85	30 (25.5, 27)		
Part-time work	22	34 (26.8, 50)		
Unemployed	105	43 (29, 50)		
Living arrangement			54.404a	<0.001
Living alone	24	51 (48.3, 54)		
Matrimonial cohabitation	162	31 (26, 42.3)		
Other	26	46 (34.8, 51.3)		
Type of stroke			0.201	0.841
Hemorrhagic stroke	33	33 (26.5, 46.5)		
Ischemic stroke	179	34 (27, 46)		
Family history			1.171	0.242
Yes	73	32 (26.5, 44)		
No	139	36 (27, 47)		
Activities of daily living			18.204a	<0.001
Poor	49	41 (27, 49)		
Average	101	40 (29, 48)		
Good	62	30 (24, 40.3)		
Combined chronic diseases			4.938a	0.176
No	43	30 (26, 43)		
1 Type	99	41 (27, 48)		
2 Types	57	34 (28, 45)		
3 Types or more	13	32 (23.5, 43.5)		
Smoking history			1.177	0.239
Yes	99	34 (27, 45)		
No	113	34 (27, 49)		
Alcohol history			0.009	0.993
Yes	99	34 (27, 46)		
No	113	34 (27, 46.5)		
Sleep			13.394a	0.001
No sleep disturbance	55	27 (24, 41)		
Suspected insomnia	123	39 (29, 47)		
Insomnia	34	41.5 (28.5, 48)		
Anxiety			28.719a	<0.001
No anxiety	92	29.5 (25, 42)		
Suspected anxiety	37	47 (39.5, 50.5)		
Definite anxiety	83	34 (29, 46)		
Depression			48.575a	<0.001
No depression	163	30 (26, 42)		
Suspected depression	32	46.5 (41.5, 49)		
Definite depression	17	47 (45.5, 52.5)		

a is H value.

### 3.3 Family functioning score of patients with stroke

The family APGAR scores of the 212 patients ranged from 2 to 10, with a mean of 6.58 ± 2.00. Each entry was scored as follows: adaptation 1.26 ± 0.75, partnership 1.27 ± 0.74, growth 1.43 ± 0.64, affection 1.45 ± 0.65 and resolve 1.18 ± 0.64. Table 3 provides the scores of the different functional groups.

**TABLE 3:** Family functioning score of first-episode stroke patients (*n* = 212).

Item	Number of cases	Family functioning score M(P_25_,P_75_)	FSS score M(P_25_,P_75_)	*H*-value	*P*-value
Severe family dysfunction group	20	3 (3, 3)	47.5 (43.5, 49.8)	146.029	<0.001
Moderate family dysfunction group	79	6 (5, 6)	47 (42, 51)		
Good family functioning group	113	8 (7, 9)	27 (24, 31)		

### 3.4 Correlation analysis of family functioning and post-stroke fatigue

Spearman’s correlation analysis was used to conduct a two-by-two correlation analysis between the scores of each entry, the total family functioning score and the FSS score of the 212 patients with stroke. The data in Table 4 reveals that PSF in patients with stroke was negatively correlated with each entry and the total family functioning score.

**TABLE 4:** Correlation analysis between family functioning and PSF (rs, *n* = 212).

Item	Adaptation	Partnership	Growth	Affection	Resolve	Total score
Fatigue severity	−0.380	−0.505	−0.470	−0.303	−0.281	−0.712
*P*	0.001	0.001	0.001	0.001	0.001	0.001

### 3.5 Multiple linear regression analysis of the post-stroke fatigue status of patients with stroke

A total of 13 variables were included, with FSS scores as the dependent variable and variables with statistically significant differences (*p* < 0.05) or correlations in univariate analysis as the independent variables. Table 5 details the assignment of the variables. Multiple linear regression analysis revealed that marital status, literacy, household income, ADLs and the family functioning score were negatively correlated influences on PSF in patients with stroke (*p* < 0.05). Based on the standardized regression coefficients and their absolute values, the degree of influence of the independent variables on PSF in patients with stroke was determined, in which the factor with the greatest influence on PSF was family functioning (β′ = −0.516), followed by marital status (β′ = −0.244), household income (β′ = −0.185), literacy (β′ = −0.181) and ADLs (β′ = −0.084) (Table 6).

**TABLE 5:** Explanatory table for the assignment of each variable.

Name	Variable	Assignment method
Gender	X2	Male = 0, Female = 1
Age (years)	X1	<50 = 1, 50∼59 = 2, 60∼69 = 3, 70∼80 = 4
Marital status	X3	Unmarried and other = 0, Married = 1
Education degree	X4	Illiterate = 1, Primary school = 2, Secondary school = 3, College and above = 4
Household income (RMB)	X5	≤2000 = 1, 2001∼5000 = 2, ≥ 5001 = 3
Medical payment methods	X6	Social security (Z1 = 1, Z2 = 0),Rural social security (Z1 = 0, Z2 = 1), Self-funded (Z1 = 0, Z2 = 0)
Employment	X7	Full-time (Z1 = 1, Z2 = 0), Part-time work (Z1 = 0, Z2 = 1), Unemployed (Z1 = 0, Z2 = 0)
Living arrangement	X8	Living alone and other = 0, Matrimonial cohabitation = 1
Activities of daily living	X9	Poor = 1, Average = 2, Good = 3
Family functioning	X10	Severe family dysfunction = 1, Moderate family dysfunction = 2, Good family functioning = 3
Sleep	X11	No sleep disturbance = 1, Suspected insomnia = 2, Insomnia = 3
Anxiety	X12	No anxiety = 1, Suspected anxiety = 2, Definite anxiety = 3
Depression	X13	No depression = 1, Suspected depression = 2, Definite depression = 3

**TABLE 6:** Multiple linear regression analysis of factors influencing post-traumatic growth in stroke patients.

Variable	β	SE	β ’	*T*-value	*P*-value
Constant	78.675	4.365	–	18.023	<0.001
Gender	−0.089	0.890	−0.004	−0.100	0.920
Age	−0.463	0.749	−0.037	−0.617	0.538
Marital status	−7.193	1.758	−0.244	−4.091	<0.001
Education degree	−2.268	0.653	−0.181	−3.473	0.001
Household income	−3.505	0.885	−0.185	−3.960	<0.001
Medical payment methods	−0.125	0.694	−0.007	−0.180	0.858
Employment	−0.141	0.712	−0.012	−0.198	0.843
Living arrangement	−0.126	1.604	−0.005	−0.079	0.937
Activities of daily living	−1.277	0.572	−0.084	−2.232	0.027
Family functioning	−8.555	0.942	−0.516	−9.084	<0.001
Frailty	6.745	1.122	0.312	6.011	<0.001
Nutritional status	−5.134	1.045	−0.274	−4.914	<0.001
Sleep	0.401	0.614	0.024	0.653	0.514
Anxiety	0.504	0.435	0.042	1.160	0.247
Depression	−0.385	0.818	−0.022	−0.471	0.638

*R*^2^ = 0.765, Δ*R*^2^ = 0.750, *F* = 49.643, *P* < 0.001.

### 3.6 Influence of frailty on post-stroke fatigue

The mean FSS scores for patients who were non-frail, pre-frail and frail were 30.12 ± 8.45, 38.54 ± 9.32 and 45.67 ± 10.21, respectively. Multiple linear regression analysis indicated that frailty (β′ = 0.312, p < 0.01) was an independent predictor of PSF. Nutritional status was assessed using the Mini Nutritional Assessment (MNA). Patients were categorized into three groups: well-nourished (MNA ≥ 24), at risk of malnutrition (MNA 17–23.5) and malnourished (MNA < 17). The results revealed that nutritional status was significantly associated with fatigue severity. The mean FSS scores for patients who were well-nourished, at risk and malnourished were 32.78 ± 9.14, 40.56 ± 10.23 and 48.33 ± 11.45, respectively. Multiple linear regression analysis demonstrated that nutritional status (β′ = −0.274, p < 0.01) was an independent predictor of PSF (Table 7). The Clinical Frailty Scale (CFS) was used to assess the frailty of patients. The CFS scores ranged from 1 (very fit) to 9 (terminally ill). Patients were categorized into three groups: non-frail (CFS 1–3), pre-frail (CFS 4–5) and frail (CFS 6–9). The results revealed that frailty was significantly associated with higher FSS scores (Table 7).

**TABLE 7:** FSS scores by frailty status.

Frailty status	Number of cases (*n*)	FSS score (Mean ± SD)
Non-frail	95	30.12 ± 8.45
Pre-frail	67	38.54 ± 9.32
Frail	50	45.67 ± 10.21
**Nutritional status**		
Well-nourished	105	32.78 ± 9.14
At risk of malnutrition	77	40.56 ± 10.23
Malnourished	30	48.33 ± 11.45

## 4 Discussion

Our findings reveal significant differences in fatigue severity among patients with stroke based on gender, age, marital status, education level, income, employment status, living arrangements, ADLs, sleep quality, anxiety and depression. The absence of significant differences in fatigue severity among patients with stroke based on the type of stroke, family history and other comorbidities suggests that addressing fatigue requires a broader, more holistic approach rather than focusing solely on clinical factors. Tailoring interventions to accommodate these socioeconomic and psychological dimensions can enhance patient care, emphasizing the need for comprehensive strategies that incorporate mental health support, lifestyle adjustments and social assistance. This integrated approach could improve patient outcomes and quality of life in post-stroke recovery.

The findings of our study demonstrate a significant negative correlation between family functioning and PSF in patients who have experienced a first episode of stroke. This relationship highlights the potential of family dynamics as a modifiable factor in the management and recovery of patients with stroke. Our results are consistent with the hypothesis that improved family support systems can significantly reduce PSF severity, which aligns with recent studies emphasizing the role of social and familial support in post-stroke recovery processes (World Health Organization, 2011; Hagan et al., 2023). This emphasizes the need for healthcare strategies that incorporate family counseling and support mechanisms as integral components of stroke rehabilitation programmes.

This study demonstrates that the level of family functioning varies widely among patients with stroke, ranging from severe dysfunction to effective functioning, with many patients falling within the low-to-medium range. This variation in family functioning highlights the importance of considering family dynamics in post-stroke care and recovery strategies. Low family functioning, characterized by poor communication, lack of emotional exchange, and insufficient mutual support, was particularly notable in areas such as partnership and resolve. These deficiencies not only hinder the patient’s emotional recovery but also exacerbate the psychological burden caused by the disease, as patients face economic pressures and changes in their roles within the family, leading to irritability, depression, and negative emotions. To address these challenges, interventions that improve family functioning are critical for enhancing post-stroke recovery. Special focus should be placed on fostering better communication and emotional support among family members. Educating family caregivers about the importance of active involvement in the patient’s recovery—such as supervising medication, diet, and rehabilitation—can strengthen family partnerships and resolve. This approach will help to alleviate the patient’s psychological burden, reduce post-stroke fatigue, and improve overall recovery outcomes. By addressing the emotional and communicative deficiencies within the family, healthcare providers can foster a supportive environment that accelerates rehabilitation and enhances the quality of life for stroke survivors. Feigin et al. (2015) also investigated this aspect, suggesting that family dysfunction and vulnerability to conflict among family members are common phenomena for patients with stroke. In this survey, family dysfunction was noted, underlining the importance of interventions to improve family functioning in patients with stroke.

Post-stroke fatigue is common and easily overlooked in patients with stroke. It occurs in 30%–72% of these patients and may persist for a long period, affecting patients’ memory and cognitive functions. It further affects the rehabilitation and long-term prognosis of patients, increasing the recurrence and mortality rates of patients with stroke; it is one of the most intolerable sequelae among these patients (Choi-Kwon et al., 2017; Lagogianni et al., 2018; Mandliya et al., 2016). The current study reveals a relatively high incidence of PSF, suggesting that healthcare providers should monitor patients who have experienced a first episode of stroke and promptly assess the presence of PSF to minimize and, if possible, avoid PSF.

This study revealed a statistically significant difference in PSF scores between patients in the good and dysfunctional family functioning groups (all *p* < 0.001); adaptation, partnership, growth, affection and resolve were negatively correlated with PSF (*p* < 0.001). Further multiple linear regression analysis demonstrated that family functioning was one of the main factors affecting PSF, suggesting that a low level of family functioning is directly or indirectly responsible for the occurrence of PSF. Stroke takes a toll on patients’ physical and psychological well-being and their family health patterns and, consequently, negatively impacts coping and recovery from the disease. The family is the strongest support system for the patient in the aftermath of stroke and largely influences the patient’s psychology. Dunbar et al. (2016) found that family functioning affects patients’ emotional and physical states, and Indelicato et al. (2020) concluded that patients with diabetes and with less family closeness had correspondingly poorer glycaemic control. The family plays a decisive role in the physical and mental health and emotional and behavioral well-being of family members (Miller et al., 2000).

Our study’s finding that improved family functioning correlates negatively with PSF corroborates the results of similar studies in the field. For instance, a study by Yang et al. observed that patients with stroke who had strong familial support exhibited significantly lower levels of PSF compared with those with minimal family interaction (Yang et al., 2021). Additionally, Ablewhite et al. reported that active family involvement in everyday care activities was associated with a faster decline in PSF symptoms over 6 months (Ablewhite et al., 2022).

Furthermore, our results demonstrated that frailty significantly impacts PSF. Patients categorized as frail had higher FSS scores than patients who were non-frail or pre-frail. This finding underscores the importance of addressing frailty in patients with stroke, particularly among the elderly, as frailty can exacerbate the overall burden of the disease and hinder recovery. Integrating frailty assessments into the rehabilitation plan can help tailor interventions that specifically address the needs of patients who are frail. This aligns with the findings that frailty significantly influences cardiovascular disease outcomes, highlighting the critical role of caregivers and family support in managing such conditions (Liperoti et al., 2021). Furthermore, nutritional status was found to be a significant predictor of PSF. Patients who were malnourished had higher FSS scores than those who were well-nourished or at risk of malnutrition. Proper nutritional support is, therefore, vital in the management of PSF. Addressing malnutrition and ensuring adequate nutritional intake can significantly enhance recovery outcomes and reduce fatigue levels in patients with stroke. This is supported by studies emphasizing the importance of nutrition in post-stroke recovery and the role of malabsorption in exacerbating fatigue symptoms (Giovannini et al., 2024; Gimovsky and Berghella, 2022).

These consistent observations across studies highlight the critical role of family dynamics in the rehabilitation process of stroke survivors. Our study adds to this body of knowledge by quantifying the strength of the relationship and emphasizing specific aspects of family functioning, such as emotional support and communication, which were found to be particularly influential. This consistency in findings across different cohorts and geographical settings suggests a robust, generalisable link between family functioning and reduced PSF, underscoring the potential benefits of integrating family-centered approaches into post-stroke care protocols.

The clinical relevance of our study lies in demonstrating how improved family functioning significantly reduces PSF in patients who have experienced a first episode of stroke. This suggests that incorporating family dynamics into stroke rehabilitation could enhance patient outcomes. Clinicians should consider family assessments and interventions, such as family therapy and education, as part of comprehensive care plans. These approaches can empower families to support the patient’s recovery effectively, potentially accelerating rehabilitation and improving their quality of life. Emphasizing family involvement in care strategies not only addresses physical recovery but also bolsters the emotional support crucial for overcoming the challenges of post-stroke adjustments.

Nonetheless, there are some limitations to this study. First, only a cross-sectional survey was conducted in the acute phase after the patients’ first stroke and when their conditions were in a stable state. Due to limited time and insufficient funds, we did not conduct a long-term and in-depth follow-up of the study participants, which resulted in an inability to assess the dynamic changes of PSF in patients who have experienced a first episode of stroke. Second, a small sample was included in this study, with only 212 valid questionnaires. Despite being an acceptable sample size, small sample sizes may limit the accuracy and reliability of the results. This may have led to limitations in the quality of statistical analyses, making it unlikely that small effects could be detected or that more refined data analyses could be performed. Third, the samples included in this study were from a single center, which limits the representativeness of the sample and leads to unlikely generalization to other regions or different types of patient populations. As a result, the external validity of the results may have been affected. For a stronger analysis of the effect of family functioning on patients who have experienced a stroke and its correlation with PSF, in future studies, multiple time points could be used as the basis for investigations to compare and analyse the dynamic changes in family functioning and PSF over time. In addition, operational and scientifically feasible nursing interventions should be developed to improve the quality of life of patients who have survived a stroke.

This study has several limitations. First, the cross-sectional design of the study limits the ability to establish causality between family functioning and post-stroke fatigue (PSF). While our findings demonstrated a significant negative association between family functioning and PSF, the study did not specifically explore whether this association was direct or indirect. It is possible that other mediating factors, such as psychological health, social support, or the patient’s physical condition, could influence the relationship between family functioning and PSF. Future longitudinal studies are needed to clarify the causal pathways and determine whether improvements in family functioning directly reduce PSF or if other factors mediate this relationship. Additionally, the sample size was relatively small and limited to a single hospital, which may restrict the generalizability of the results. The study also focused on patients during the acute phase of stroke recovery, and long-term follow-up was not conducted to assess the dynamic changes in family functioning and PSF over time. Future research should aim to establish whether the relationship between family functioning and PSF is direct or mediated by other factors, such as psychological health, social support, or physical comorbidities. Longitudinal studies are needed to explore the dynamic changes in family functioning and PSF over time, particularly during the chronic phase of stroke recovery. Additionally, future studies could examine the effectiveness of specific family-based interventions—such as family therapy, caregiver training, and psychosocial support programs—in reducing PSF and improving patient outcomes. Expanding the study to include more diverse populations and settings could also help generalize the findings and identify culturally appropriate interventions. Finally, exploring the interaction between family functioning, frailty, and nutritional status may provide deeper insights into comprehensive care strategies for stroke survivors.

## 5 Conclusion

This study demonstrates a significant negative correlation between family functioning and post-stroke fatigue (PSF), indicating that patients with better family dynamics experience less severe PSF. Beyond the role of family functioning, factors such as marital status, household income, education level, and activities of daily living also contribute to the severity of PSF. These findings suggest that comprehensive rehabilitation strategies should incorporate family-centered approaches to improve not only physical recovery but also the emotional and psychological well-being of stroke survivors. By enhancing communication, emotional support, and practical assistance within the family unit, healthcare providers can help reduce the burden of PSF and promote better recovery outcomes. Furthermore, targeted interventions addressing socioeconomic and educational disparities may help mitigate the impact of PSF in certain populations

## Data Availability

The original contributions presented in the study are included in the article/supplementary material, further inquiries can be directed to the corresponding author.
